# Optimising Extraction of Specific Food Allergens from Challenging Food Matrices for Immunoassay Quantification

**DOI:** 10.3390/foods14203501

**Published:** 2025-10-14

**Authors:** Max D. Bermingham, Rhys T. Meredith, Hayley Mills, Sarah Maddocks, Martin D. Chapman, James A. Blaxland, Maria A. Oliver

**Affiliations:** 1 InBio, Cardiff CF23 8HA, UK; 2 Cardiff School of Sport and Health Sciences, Cardiff Metropolitan University, Cardiff CF5 2YB, UK; 3 InBio, Charlottesville, VA 22903, USA

**Keywords:** food allergen, extraction, multiplex array, immunoassay, food matrices, chocolate, thermal processing

## Abstract

Simultaneous extraction of multiple clinically relevant, specific allergens from complex, processed foods for immunoassay quantification remains challenging. Moreover, shared extraction buffers remain elusive, which limits the effectiveness of multiplex (multi-target) technology. This study aimed to optimise extraction of specific allergens from challenging food samples and identify shared methods of extraction for multiplex analysis. Incurred processed food matrices (chocolate dessert, raw and baked biscuit) were prepared and extracted with 10 different buffers varying in base, pH, and additive content. Extracts were analysed by allergen-specific multiplex array and ELISA. Optimised recovery of 14 food allergens was obtained from complex incurred matrices using two extraction buffers: 50 mM carbonate bicarbonate with 10% fish gelatine, and PBS with 2%-Tween, 1 M NaCl with 10% fish gelatine and 1% PVP. In most cases, optimised buffers provided 50–150% recovery from incurred foods. Matrices that contained chocolate or were subject to thermal processing had lower recoveries. Optimised allergen extraction methods can be used to analyse foods associated with treatment and prevention of allergy and reference materials for clinically relevant allergen content and identify allergen contamination. Identification of shared, optimised extraction buffers will result in increased sample throughput in multiplex immunoassay techniques.

## 1. Introduction

Food allergen analysis is crucial in protecting allergic consumers from undeclared allergen presence (UAP). However, the complexities of food allergen analysis have been well documented. Food processing and the presence of interfering compounds including but not limited to salts, fats, and polyphenols in samples containing cocoa are known to interfere with food allergen extraction, potentially leading to an under-estimation of allergen content, and potential false negatives in identifying allergen presence [1].

Numerous studies have reported methods for extracting food allergens from challenging matrices, including the impact of extraction times, temperatures, and extraction buffer composition on improving allergen recovery [2,3,4,5,6,7]. Extraction buffer formulation changes resulted in significant improvement to allergen recovery. Changes to buffer pH, salt, and detergent content, and presence of protein- and non-protein-blocking additives such as non-fat dry milk (NFDM), fish gelatine (FG), and polyvinylpyrrolidone (PVP), or the use of ‘denaturing buffers’, yielded notable improvements [1,3,5,6,8,9,10]. It is believed these additives increase solution ionic strength and disrupt matrix interactions to release and solubilise allergen, thus available for immunoassay quantification [1,3,4,6,8,11,12,13,14,15,16,17,18,19,20,21,22,23]. For additional information on buffer components, see Supplementary Material Section S1. Whilst some common ground between extraction methods exists, a ‘universal’ allergen extraction method suitable for extracting multiple allergenic proteins remains elusive.

Total protein enzyme-linked immunosorbent assays (ELISAs) have been the ‘go to’ method for food allergen analysis for decades, given their robustness, ease of use, relative low cost, and quantitative readout. These methods use polyclonal antibodies (pAbs) raised against allergenic commodity extracts and are calibrated with extracts of allergen sources [24,25]. These methods have proved instrumental in identifying UAP [26,27,28]. However, they hold a number of limitations due to the use of polyclonal antibodies and crude allergen source calibrators, including ambiguity over the analytical target, a lack of harmonisation between kit manufacturers, risks of cross-reactivities and batch–batch variation [25,29].

To address these challenges, allergen-specific immunoassays (predominantly ELISAs) have been developed that measure the clinically relevant proteins from allergen sources that are known to cause allergic responses [30]. Allergen-specific immunoassays utilise purified component allergens for standards and for immunisation and generation of allergen-specific mono-/polyclonal antibodies. Examples of allergen-specific ELISAs are well documented in the literature, highlighting the benefits of allergen-specific measurement and molecular targeting which include improved relevance of measurement, specificity, standardisation, accuracy, and reporting clarity compared to total protein ELISA [11,30,31,32,33,34]. Clinically relevant proteins often have high abundance, present strong thermal and digestion resistance, and so show potential as excellent markers for contamination assessments [30,35,36,37]. Additionally, allergen-specific methods have been used to assess foods associated with the treatment or prevention of allergy, e.g., oral food challenges (OFCs), early introduction foods (EIFs) and oral immunotherapy (OIT) [8,11,38,39,40,41]. A key challenge for specific allergen measurement in foods by immunoassays lies in the limited information on optimised extraction from multiple matrices. Previous data on allergen extraction optimisation focuses on the use of total protein kits. Few specific extraction optimisation and quantification have been performed, where the majority of these studies have not determined percentage matrix recovery relating to a source material and only investigated a single food matrix [3,6].

The need for universal extraction methods becomes apparent when considering multiplex allergen analysis. The US FDA successfully demonstrated the potential of multiplex immunoassay detection of food allergens with the xMAP Food Allergen Detection Assay (FADA), capable of detecting 15 allergenic sources in a single test. This was a promising leap forward in the analytical community, yet still holds challenges limited by allergen extraction, with results suggestive that a maximum recovery value of 20% from chocolate and 60% from baked muffins was achieved [42,43]. To improve recovery, several more extractions may be necessary, at which point the time- and cost-saving benefits of multiplex analysis are called into question since several plates may be required to analyse a given sample. The multiplex immunoassay approach for food allergen detection has been developed further by utilising allergen-specific immunoassays and purified allergen calibrants to create ‘MARIA for Foods’, a multiplex methodology with benefits of improved standardisation, specificity, and reporting clarity [33].

In the present study, optimised extraction conditions for 14 specific allergens covering 11 major allergen sources from complex, incurred food matrices and paired allergen source materials were assessed using an allergen-specific multiplex array and ELISA. Incurred chocolate dessert, biscuit dough, and baked biscuit matrices were prepared in-house and allergen extraction was optimised through testing different extraction buffers, with varying pH, salt content, and addition of additives (fish gelatine, PVP, and NFDM). The aims were to (i) increase the understanding of how specific allergens are impacted by different matrices and by food processing and (ii) to identify shared methods of allergen extraction covering multiple matrix types and allergen sources to fully realise the potential of multiplex allergen-specific detection.

## 2. Methods and Materials

### 2.1. Extraction Buffer Preparation

The composition of the extraction buffers used in this study are documented in Table 1. Candidate extraction buffers were selected from literature research and previous unpublished data [1,3,5,6,8,9,10].

### 2.2. Preparation of Incurred Foods

Food matrices chosen were a chocolate dessert matrix which was based on the Europrevall matrix [44], and a rice-flour-based biscuit dough that would be analysed raw and baked. Placebo matrices containing no allergen were prepared initially, serving as an analytical negative control and a base for the preparation of incurred samples. Chocolate dessert and biscuit dough were incurred with allergen source material flour/powders (Appendix A). The protein content of allergen source materials determined by Kjeldahl analysis (using the standard nitrogen conversion factor of 6.25) was used to incur food matrices with defined levels of total allergenic protein of 10, 100, and 1000 ppm. Following incurring, biscuits were baked in 40 g portions of dough at 185 °C for 15 min. Additional information on allergen source materials and food matrix preparation can be found in Appendix A and Supplementary Material S2, Tables S1–S3, respectively.

### 2.3. Allergen Extraction

Extraction buffer was added to samples in 1:10 sample/buffer ratio (e.g., 1 g sample/10 mL extraction buffer), vortex mixed for 30 s, and incubated for 15 min in an orbital incubator (Stuart SI500, Staffordshire, UK) set to 60 °C shaking at 175 rpm. Following extraction, the supernatant was obtained by centrifugation at 1250 rcf, 20 min at 4 °C. Clarified supernatant containing extracted protein was taken from the middle to avoid sampling separated insoluble material.

### 2.4. Optimising Extraction of Specific Allergen from Allergenic Source Materials

A ‘source material mix’ (SMM) was prepared from single food source materials (flours or powders from egg, milk, peanut, soy, cashew, walnut, almond, hazelnut, sesame, celeriac [celery], and shrimp) which contained defined amounts of specific allergens. For further information on the source materials used and SMM preparation, see Supplementary Table S3. Triplicate extractions from the SMM were performed using the different extraction buffers and analysed by allergen-specific multiplex array, or ELISA. Allergen content measured in SMM was used to determine recovery from incurred foods (Section 2.5).

### 2.5. Optimising Extraction from Incurred Foods

Initial recovery optimisations from incurred foods (prepared in Section 2.2) involved assessing and optimising recovery from the lowest dose incurred samples, 10 ppm, as this would present the biggest challenge in the study. Where allergen could not be detected at the 10 ppm level (Api g 1), initial optimisations were made using the high dose 1000 ppm. Triplicate extractions from the incurred foods were performed using the different extraction buffers and analysed by allergen-specific multiplex array or ELISA.

### 2.6. Allergen Quantification by Immunoassay

#### 2.6.1. MARIA for Foods (Multiplex Immunoassay)

MARIA for Foods (InBio, Charlottesville, VA, USA) multiplex array was used to measure Ara h 3, Ara h 6, Bos d 5, Bos d 11, Gal d 2, Ana o 3, Jug r 1, Pru du 6, Ses i 1, Api g 1, and shrimp tropomyosin. The assay utilises Luminex xMAP bead-based assay technology (Luminex Corporation, Austin, TX, USA), performed as previously described [8,33,45]. Unique bead sets (distinguishable by fluorophore signal) with covalently coupled allergen-specific mAb/pAb for each respective allergen target were used for allergen capture. Highly purified allergen proteins were used as assay standards and incubated with capture beads alongside food extract samples. Beads were washed and subsequently incubated for 60 min with biotinylated detection antibodies. Beads were washed and incubated with a fluorescent probe, streptavidin-phycoerythrin (AbCam, Cambridge, UK. Cat No. AB239759) (30 min), to allow for the detection of bound allergen/detection antibody sandwich. A Bio-Plex 200 instrument (Bio-Rad, Hertfordshire, UK. Cat No. 171000205) was used to measure the mean fluorescent intensity of bead sets (50 beads per allergen, per sample), and the concentration of allergen in food extract samples interpolated from purified allergen standard curves with Bio-Plex Manager software (Bio-Rad, Hertfordshire, UK. Ver 6.2.0.175).

#### 2.6.2. Enzyme-Linked Immunosorbent Assay (ELISA)

ELISAs were performed to measure Gal d 1, Gly m 5, and Cor a 9, and performed as previously described [19,38] using mAbs and purified allergen standards from InBio, Charlottesville, VA, USA. Briefly, allergen-specific mAb were used for allergen capture in a pre-coated NUNC-MAXISORP (ThermoFisher Scientific, Rochester, NY, USA. Cat No. 439454) 96-well plate. Highly purified allergen proteins were used as assay standards. Bound standard and samples were detected using biotinylated monoclonal antibodies and streptavidin-peroxidase (Sigma-Aldrich, Saint Louis, MO, USA. Cat No. S5512), or polyclonal antibody and goat anti-rabbit peroxidase conjugate (Jackson ImmunoResearch, West Grove, PA, USA. Cat No. 111-036-046). BioFX TMB substrate (Surmodics, Eden Prairie, MN, USA. Cat No. TMBS-1000-01) was added for detection of enzyme conjugate, and the reaction was stopped by addition of 0.5 N sulfuric acid (Fisher Scientific, Loughborough, UK. Cat No. J/8440/17).

### 2.7. Statistics

Statistical analysis was performed using GraphPad Prism software (GraphPad, Boston, MA, USA. Ver 10.2.2). One-way Anova with Dunnett’s multiple comparison was used to identify significant differences in yield from source material extractions. Dunnett’s multiple comparison test is the recommended statistical test for assessing significant differences from an assigned control (i.e., extraction method with greatest yield) where there are multiple groups. The extraction method yielding the highest amount of allergen was assigned the ‘control’ from which to compare significant differences.

### 2.8. Calculation of Allergen-Incurred Matrix Recovery

For allergen source materials, the optimised specific content was divided by the total protein content previously determined by Kjeldahl to calculate an expected amount of specific allergen per ppm of total protein. This value was multiplied by the incurring dose (e.g., 10 ppm). An example calculation is included in Supplementary Information, Section S3.

## 3. Results

### 3.1. Extraction Optimisation of Allergen Source Materials

Source materials were assessed for allergen content following extraction in different extraction buffers (Table 2). Allergen yield from source material varied >900-fold depending on the extraction buffer used, mostly attributed to the majority of allergens presenting very low extraction yield by use of buffer C. The extraction method resulting in the highest allergen yield was used as a benchmark from which recovery and significance were assessed against other extraction methods tested. Allergen content measured in the spike mix was calculated to that expected from the source material.

Optimal extractions from source materials required the use of different extraction buffers, depending on the allergen of interest. Buffer A provided optimal extraction for 8 of the 14 allergens analysed (Pru du 6, Jug r 1, Ana o 3, Ara h 3, Ara h 6, Gal d 2, Bos d 5, and Gly m 5). Buffer D resulted in optimal extraction of Bos d 11, with all other buffers extracting significantly less allergen. Buffer D also proved optimal for extraction of Gal d 1. For Cor a 9, buffer F was optimal, with buffers A and D also providing excellent recovery (91%), with differences in results between the three buffers not being statistically significant (*p* > 0.05). For Ses i 1 and STM, buffer E was optimal, whilst buffer A for Ses i 1 and buffer D for STM also gave excellent recoveries (95% and 92%, respectively).

Buffer choice for Api g 1 was dependent on the extraction temperature. Buffer E was optimal when performing extractions at 60 °C (201 µg/g); however, when extraction temperatures were reduced to 37 °C, the greatest yield of Api g 1 was obtained with buffer A (330 µg/g).

Given the small, non-significant differences between results for Cor a 9, Ses i 1, and STM from the ‘optimal’ buffer versus either buffers A or D, the results demonstrate that all 14 allergens can be extracted from the SMM with 90–100% recovery from just two extraction buffers (buffers A and D).

### 3.2. Calculation of Extracted Specific Allergen Abundance

Using the optimised extracted specific allergen and total protein, as determined by Kjeldahl analysis, a percentage abundance of specific allergen for each source material was determined (Table 3). Extracted specific allergens represented 0.2–62% abundance of total protein quantified by Kjeldahl analysis, supporting previous studies that identify specific allergens as major extractable protein fractions of allergen sources [46,47,48,49,50,51,52,53,54,55,56]. The optimised specific allergen recovery values (Table 3) were used as a calibrating value to estimate expected allergen content from incurred foods (Section 2.8).

### 3.3. 10 ppm Recovery Optimisation

**Figure 1:**
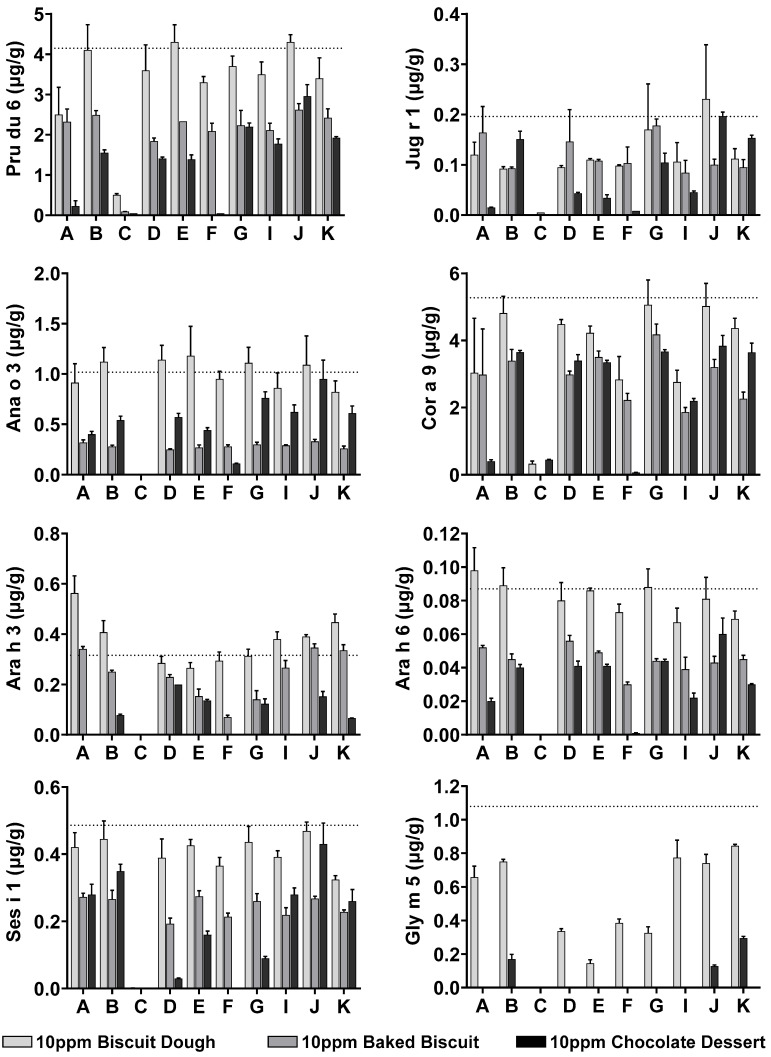
Extraction optimisation of Pru du 6, Jug r 1, Ana o 3, Cor a 9, Ara h 3, Ara h 6, Ses i 1, and Gly m 5 from 10 ppm incurred biscuit dough (light grey), baked biscuit (dark grey), and chocolate dessert (black). See Table 1 for formulation of extraction buffers A–K. Expected specific allergen content at 10 ppm based on source material allergen content (Table 2) denoted by dashed line. Error bars represent standard deviation of triplicate extracts.

**Figure 2:**
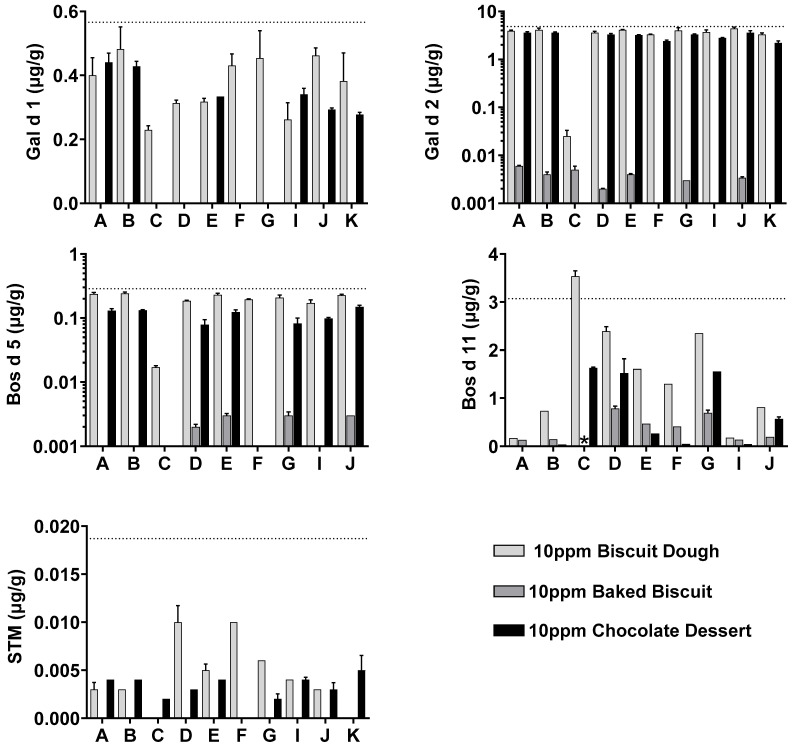
Extraction optimisation of Gal d 1, Gal d 2, Bos d 5, Bos d 11, and shrimp tropomyosin (STM) from 10 ppm incurred biscuit dough (light grey), baked biscuit (dark grey), and chocolate dessert (black). See Table 1 for formulation of extraction buffers A–K. Expected specific allergen content at 10 ppm based on source material allergen content (Table 2) denoted by dashed line. Milk allergens not analysed for samples extracted in buffer K as buffer contained milk protein. * Sample positive, but not quantifiable. Error bars represent standard deviation of triplicate extracts.

The 10 ppm incurred complex foods were assessed for allergen content by use of different extraction buffers. Analyses indicated that extractions from baked biscuit and chocolate dessert had a lower allergen yield when compared to biscuit dough. With the exception of Api g 1, all allergens were detectable at the 10 ppm dose level in incurred biscuit dough and chocolate. Gal d 1, STM, and Gly m 5 were no longer detectable at the 10 ppm level in the baked biscuit by use of any of the extraction methods trialled. Figure 1 and Figure 2 document specific allergen measurement for all allergens (with the exception of Api g 1). Upper dashed lines denote the expected level of specific allergen at 10 ppm, based on optimised extracted values from Table 3.

Incurred sample analysis at 10 ppm confirmed the hypotheses that extraction buffer selection is crucial for optimised allergen detection, depending on the matrix under investigation. Optimal extraction from allergen source materials was obtained by use of buffer A for the majority of allergens tested (Section 3.1). However, when allergen was incurred into complex food matrices, this no longer proved optimal for any of the allergens analysed. Instead, alternative extraction buffers B, D, G, and J with different buffer-base, salt content, and presence of additives including fish gelatine (FG), BSA, NFDM, and PVP were required to improve extraction. Buffer J demonstrated strong extraction yield in the chocolate sample when compared to buffer A, with the addition of FG and PVP resulting in a 2–26-fold increase in detectable allergen for Pru du 6, Jug r 1, Ana o 3, Cor a 9, Ses i 1, Ara h 3, and Ara h 6. Egg allergen Gal d 2 and cow’s milk allergen Bos d 5 demonstrated similar allergen yields with several extraction methods. Buffer J improved the extraction of Bos d 5 from the chocolate matrix to >50%, albeit a marginal improvement from 45 to 46%.

Optimal recovery of Bos d 11 from incurred foods was obtained by buffers C, D, and G; however, buffer C provided questionable quantification data. At low dilutions (e.g., 1:10), samples extracted in buffer C presented strong evidence of assay interference for all matrix types. For analyses of contamination less than 10 ppm, this would cast doubt over any results obtained as they would likely be under-estimated. Moreover, for the baked biscuit sample, buffer C resulted in a poor dilution linearity, meaning the sample could not be reliably quantified. This observation was repeated across three plates, covering six sample extraction replicates; see online repository for information, Table S7. Given the denaturing and reducing properties of buffer C, it is plausible that residual buffer in the sample interferes with antibody binding by disrupting antibody or antigen structure or preventing non-covalent interactions. Due to this, and the low yield observed from source materials (Section 3.1) and incurred foods across the board, it was decided that buffer C was not to be used for further analyses.

Review of the dataset identified A, B, D, G, J, and K as candidate-optimised extraction buffers, and they were taken forward for analysis of 100 and 1000 ppm incurred samples.

### 3.4. Api g 1 Extraction Optimisation

Optimisation of Api g 1 extraction in incurred foods was based around a 1000 ppm dose due to being undetectable at 10 ppm. The refined list of extraction buffers identified in Section 3.2 was used. Initial investigations quantified extracts at 60 °C, with the intention to take optimised buffer forward for extraction at the lower temperature of 37 °C. Interestingly, 60 °C extractions did not negatively impact Api g 1 detection when present in complex foods, opposing findings observed in Section 3.1 for source materials (Supplementary Table S4). Subsequently, all extractions of Api g 1 in complex foods were performed at 60 °C. Summarising data for Api g 1 is included in Section 3.5 for multi-dose analysis.

### 3.5. Multi-Dose Recovery Analysis at 10, 100, and 1000 ppm

**Figure 3:**
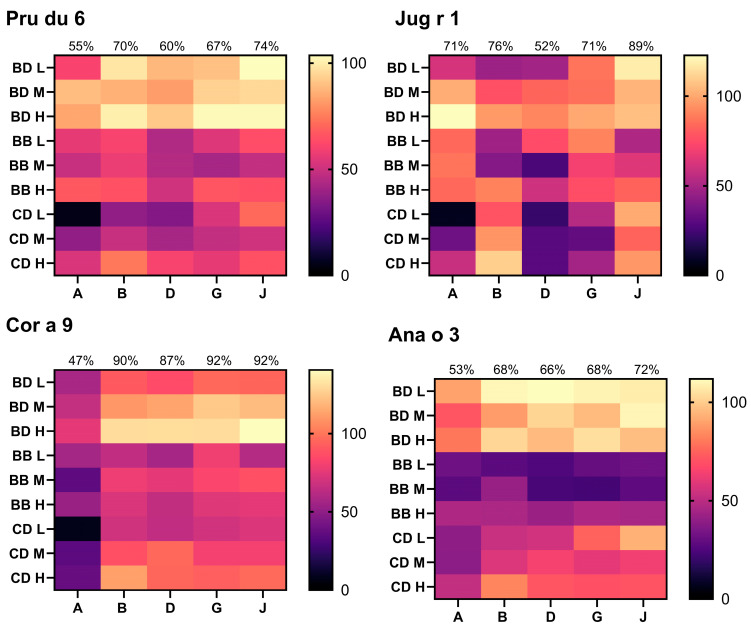
Multi-dose recovery analyses for Pru du 6, Jug r 1, Cor a 9, and Ana o 3. Percent matrix recovery from source material plotted for biscuit dough (BD), baked biscuit (BB) and chocolate dessert (CD). Results from 10 ppm (L), medium 100 ppm (M), and high 1000 ppm (H) material are shown. Extraction method used denoted by buffer letter identifier on x-axis. See Table 1 for buffer composition. Average % recovery plotted at the top of each column. Results below LOD were assigned a value of 0%.

**Figure 4:**
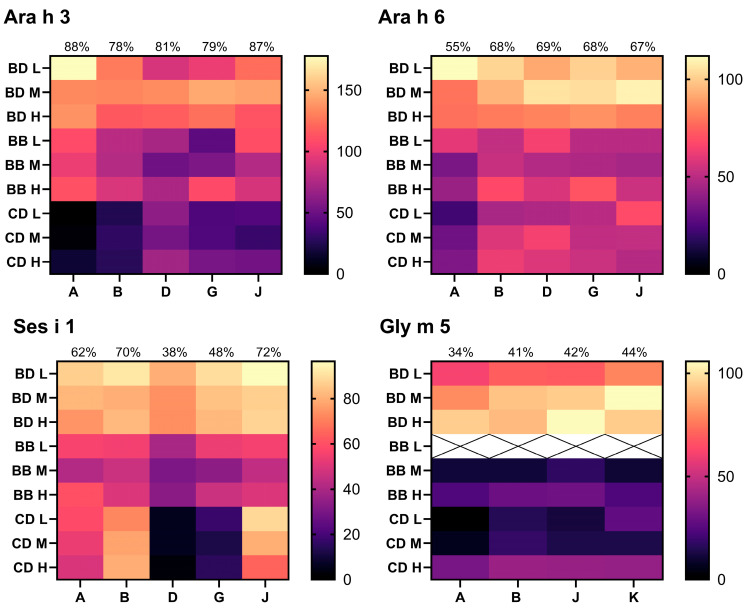
Multi-dose recovery analyses for Ara h 3, Ara h 6, Ses i 1, and Gly m 5. Percent matrix recovery from source material plotted for biscuit dough (BD), baked biscuit (BB) and chocolate dessert (CD). Results from 10 ppm (L), medium 100 ppm (M), and high 1000 ppm (H) material are shown. Extraction method used denoted by buffer letter identifier on x-axis. See Table 1 for buffer composition. Crossed panels denote result below method limit of detection. Average % recovery plotted at the top of each column. Results below LOD were assigned a value of 0%.

**Figure 5:**
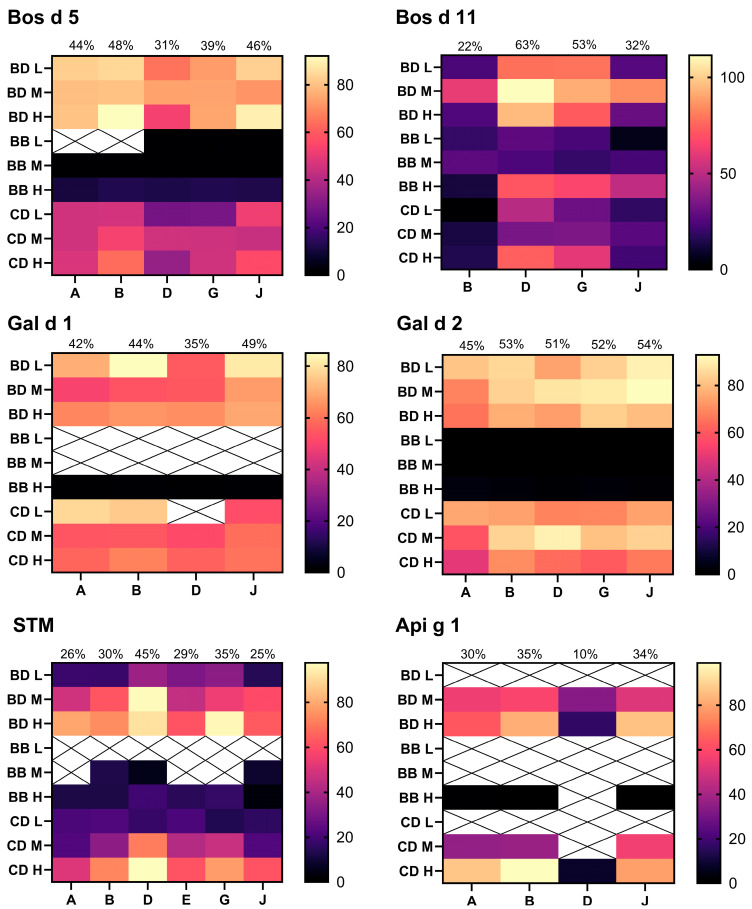
Multi-dose recovery analyses for Bos d 5, Bos d 11, Gal d 1, Gal d 2, shrimp tropomyosin (STM), and Api g 1. Percent matrix recovery from source material plotted for biscuit dough (BD), baked biscuit (BB), and chocolate dessert (CD). Results from 10 ppm (L), medium 100 ppm (M), and high 1000 ppm (H) material are shown. Extraction method used denoted by buffer letter identifier on x-axis. See Table 1 for buffer composition. Crossed panels denote result below method limit of detection. Average % recovery plotted at the top of each column. Results below LOD were assigned a value of 0%.

Extraction buffers A, B, D, E, G, J, and K identified from 10 ppm analyses were used for analysis of 100 and 1000 ppm samples to build recovery data over multiple dose levels. Heatmap Figure 3, Figure 4 and Figure 5 show the recovery for each sample and extraction buffer relative to the expected specific allergen content from source material extraction optimisation (Section 3.1). The following comments relate to Figure 3 and Figure 4, and unless otherwise stated, reflect the average recovery across all three dose levels (10, 100, and 1000 ppm) and three matrices. Where a recovery value is termed ‘acceptable’, this refers to within 50–150%, as recommended by the Association of Official Analytical Collaboration International (AOACI) [58].

Allergens from tree nuts (Pru du 6, Jug r 1, Ana o 3, Cor a 9), sesame (Ses i 1), egg (Gal d 1 and Gal d 2), and celery (Api g 1) presented the strongest recoveries by use of extraction buffer J. Using buffer D, peanut allergens Ara h 3 and Ara h 6 presented acceptable recoveries of 50–150%. However, looking at the results from the different matrices, buffers A and J presented a greater yield of Ara h 3 from incurred biscuit samples (92–149%), yet recovery from the chocolate-based matrix was poor (7–42%). Ara h 6 presented comparable matrix recovery by use of buffer D and J, with average recovery across all three doses, and matrices being 69% and 67%, respectively.

Milk allergens Bos d 5 and Bos d 11 required separate extraction buffers that could not be used interchangeably for acceptable recoveries within 50–150%. Bos d 5 presented the strongest recoveries with buffers B and J, with both methods yielding average recovery across all dose levels of 80% and 50% recovery in biscuit dough and chocolate dessert, respectively. However, it is noteworthy that buffer J allowed for detection of 10 ppm milk protein in the baked biscuit, and greater recoveries in 10 ppm chocolate. The greatest yield of Bos d 11 recovered from incurred foods was obtained by buffer D (95% and 52% recovery in biscuit dough and chocolate dessert). The greatest yield of tropomyosin was also obtained by use of buffer D. Optimal yield of Gly m 5 was obtained by buffer K; however, results were only marginally higher than those obtained with buffers B or J (<5% on average). Practically, buffer J is more suitable than buffer K as it is also optimal for analysis of several other allergens as described above and so will improve efficiency when analysing samples for several allergens.

In summary, extraction buffers D and J were deemed as optimised extraction buffers for the analysis of 14 specific allergens from peanut, milk, egg, cashew, hazelnut, walnut, almond, soy, sesame, celery, and shrimp.

Prior to the extraction study, the placebo food matrix samples were extracted and analysed using buffer A to confirm allergen-free status. Buffer optimisation resulted in increased allergen yield, and so placebo samples required extraction and analysis using the optimised extraction buffers D and J. Interestingly, using the optimised extraction buffer J for Pru du 6 identified trace levels of almond contamination in biscuit dough and baked biscuit (0.012/0.007 µg/g Pru du 6, respectively). These trace values were subtracted from allergen measurements of 10, 100, and 1000 ppm samples prior to calculating matrix recovery. Summarising recovery values for the optimised extraction buffers D and J are presented in Table 4.

When considering all three dose levels, with the exception of Api g 1, all allergens presented an acceptable average recovery from biscuit dough ranging from 50 to 150% and were detectable at 10 ppm. Similarly, acceptable average recoveries of 50–150% were obtained for the majority of allergens analysed from chocolate dessert and detectable at all doses, with the exception of Gly m 5, which had low matrix recovery of 22%, and Api g 1, which could not be detected at 10 ppm.

Baked biscuit analysis presented lower recoveries than for raw biscuit dough, suggesting all allergens are impacted by heating to varying extents. Acceptable recoveries of 50–150% were obtained for Ara h 3, Ara h 6, Pru du 6, Jug r 1, Cor a 9, and Ses i 1, and all other allergens had recovery < 50%. Despite Gal d 2, Bos d 5, Bos d 11, and Ana o 3 recoveries being low, these allergens were still successfully detected at the 10 ppm level.

## 4. Discussion

Advancements in food allergen analysis in recent years have aimed to develop mAb-based allergen-specific immunoassays that target the clinically relevant component allergens in foods, and to detect multiple target allergens simultaneously. Such methods show potential for improvements in standardisation of allergen measurement with defined analytical targets and testing throughput [33,42,43,59]. Key challenges remain for the utilisation of these improvements in that (i) minimal information is published on which buffers are most suitable for the extraction of specific allergens from foods, and (ii) there is no universal extraction methodology covering all allergen sources. Performing several extractions on one sample therefore reduces the efficiency of multiplex allergen analysis. The present study optimised the extraction of specific allergen proteins for immunoassay quantification and assessed whether common extraction buffers for multiplex food allergen analysis could be identified.

This study focused on optimising extraction buffer composition to improve extraction yield. Previous internal studies [unpublished] on extraction times (15, 30, 60, and 120 min) and sample/extraction buffer ratio (1:10, 1:20, and 1:40) did not yield notable differences in peanut allergen recovery from peanut flour and chocolate incurred with peanut. Extraction temperature at 60 °C compared to room temperature (RT) and 40 °C improved allergen recovery. Major improvements were obtained by use of different extraction buffers. Similar findings were observed by Filep and Chapman when optimising extraction of a number of specific allergens (Ana o 3, Ara h 3, Ara h 6, Bos d 5, Bos d 11, Cor a 9, Gal d 1, Gal d 2, Gly m 5, and shrimp tropomyosin) from source materials [8]. Extractions at 60 °C demonstrated a greater yield in detectable allergen, or at least no negative impact over extraction at RT. No data could be found in the literature on the impact of 60 °C extractions compared to RT for Pru du 6, Jug r 1, Ses i 1, and Api g 1, and so were assessed in a small-scale study. These data confirmed that extraction was improved or not negatively impacted by extracting at 60 °C for Pru du 6, Jug r 1, and Ses i 1 (Appendix B). However, Api g 1 recovery from source material was reduced by approximately 50% when compared to RT or 37 °C extraction. Interestingly, detection of Api g 1 in incurred foods was not impacted at 60 °C compared to 37 °C, which could suggest protective effects from complex food matrices (see Supplementary Information Section S4, Table S4). Extractions beyond 60 °C were not tested due to proteins Bos d 5 (cow’s milk) and Api g 1 (celery) having denaturation temperatures close to this [60,61]. Moreover, previous research investigating total protein extraction from almond identified a 60 °C extraction to be optimal over lower temperatures; however, when this was increased to 70 °C, a decline in yield was observed, due to protein denaturation and aggregation [62]. These previous findings justify the rationale behind the physical extraction technique used in the present study, sample/extraction buffer ratio of 1:10, extracted for 15 min at 60 °C.

The results from the present study demonstrate the necessity and complexities of optimising buffer composition for food allergen extraction. Here, optimised methods of extraction have been identified for analysing allergens from whole allergen source commodities and example complex foods. The dataset obtained herein identified that, overall, 2–3 extraction buffers provided optimal, or acceptable (50–150%), recovery for 14 specific allergens from peanut, milk, egg, cashew, hazelnut, walnut, almond, soy, sesame, celery, and shrimp tropomyosin from three complex incurred food matrices and paired allergen source materials using allergen-specific immunoassays. These buffers were buffer A (PBS, 2%-Tween, 1 M NaCl, pH 7.4), buffer J (PBS, 2%-Tween, 1 M NaCl, 10% FG, 1% PVP pH 7.4) and buffer D (50 mM sodium carbonate/bicarbonate buffer with 10% fish gelatine, pH 9.6). The finding that two optimised extraction methods can cover the analysis of 14 major allergen proteins from complex foods will improve both effectiveness and accuracy of allergen-specific multiplex food allergen analysis.

Buffers A and D provided optimal yield from source materials, whereas from complex, incurred foods, two extraction buffers, D and J, yielded strong allergen recovery. In most cases, recovery was to within 50–150% of expected when considering all doses and matrices. Buffer J provided optimal recovery of Pru du 6, Jug r 1, Ana o 3, Gal d 1 Gal d 2, Bos d 5, Gly m 5 and Api g 1, Cor a 9, and Ses i 1. Buffer J is a variation of buffer A that includes fish gelatine and PVP additives, indicating additives are required in the core buffer formulation in complex foods. The most notable improvements in extraction efficiency using buffer J were obtained from chocolate samples, where 2–26-fold increases in detectable allergen were observed for Pru du 6, Jug r 1, Ana o 3, Cor a 9, Ses i 1, Ara h 3, and Ara h 6. Buffer D provided optimal recovery of Bos d 11 and STM from all three incurred matrices. Milk allergens Bos d 5 and Bos d 11 demonstrated the complexities of food allergen detection, in that allergens of the same source origin required separate extraction buffers that cannot be used interchangeably for acceptable recoveries within 50–150%. Peanut allergens demonstrated that complex food matrices may require different extraction buffers for optimal yields. For example, for optimal analysis of Ara h 3 in biscuits and chocolate, it could be argued that two extraction methods should be used; D for chocolate and J for biscuits, as yields were higher for these buffers in each respective matrix. However, one must consider that when applying these methods to analysis of real foods, matrices may not be so well defined and cross-overs could exist (e.g., chocolate biscuits). Buffer D, therefore, may be better suited for use as an ‘all round’ extraction method for peanut. These results also suggest each new food matrix should undergo preliminary extraction assessment with several extraction buffers.

Low recoveries and assay interference were observed when analysing samples extracted in buffer C. This buffer is a reducing and denaturing buffer containing 0.1 M Tris, 1% SDS, and 0.1 M sodium sulphite [9]. The use of denaturing buffers has been successful in the analysis of processed foods in detecting denatured allergen. For denaturing buffers to be suitable in immunoassay analysis, the method itself must be capable of recognising denatured allergen protein [9,63]. The immunoassay methods used herein utilise antibodies raised against purified allergens in their native state. It is therefore plausible that the immunoassays recognise conformational epitopes, rather than are linear and are not suitable for this type of analyses. At low sample dilutions, it is probable that assay interference may be observed due to residual extraction buffer interfering with antibody binding by disrupting antibody or antigen structure, or preventing non-covalent interactions, since it is known that SDS (an anionic detergent) is capable of disrupting non-covalent antibody–antigen complexes [63,64,65]. Interestingly, samples extracted in buffers containing a different detergent, Tween-20, did not present such interferences. Tween-20 is commonly used in ELISA and xMAP assay running buffers, so is largely compatible with antibody–antigen incubations. Tween-20 is a non-ionic detergent and so does not impact native conformation like other anionic detergents, such as SDS, but has efficacy in breaking apart non-specific interactions [17]. Tween-20 has been reported to allow for the solubilisation of membrane proteins, and demonstrates capacity to breakdown lipid matrices [18]. Supporting information on interference, or lack of, for buffers containing SDS and Tween is found in Supplementary Information Tables S6 and S7.

All allergens in the study appeared impacted by heat treatment, with lower recoveries observed across the board when compared to the matched biscuit dough. Following baking, strong recoveries of 50–150% were obtained for Ara h 3, Ara h 6, Pru du 6, Jug r 1, Cor a 9, and Ses i 1, and all other allergens had recovery < 50%. Whilst Gal d 2, Bos d 5, Bos d 11, and Ana o 3 recoveries were low, allergens were still detectable at the 10 ppm level and so demonstrate the effectiveness of combined extraction and sensitive assay methodology. Interestingly, Bos d 5 and Bos d 11 recovery was acceptable at the higher 1000 ppm dose, which may suggest heat-protective effects at higher levels of allergen. The reason behind this is unclear. For Bos d 11, hypotheses can be made based on allergen form. Caseins exist in their native matrix as a colloidal casein micelle structure, where Bos d 9, Bos d 10, and Bos d 11 form a hydrophobic core surrounded by a peripheral hydrophilic layer of Bos d 12, bridged by colloidal calcium phosphate [66,67]. Casein micelles can be disrupted by changes in pH, shearing, salts, and dissolving of colloidal calcium phosphate [66,67]. One could speculate that these factors may have a more pronounced effect at lower dose-incurring levels, meaning Bos d 11 is no longer present within a micelle structure, which may be providing protection to Bos d 11 during processing.

The mechanism of why a lower amount of allergen was detected in the baked sample remains unknown and could be investigated in future studies. Plausible explanations include the loss of conformational IgG-binding epitopes due to heat denaturation, epitope masking from Maillard reactions, or protein aggregation and/or precipitation as a result of heat treatment [68]. Mass spectrometry analysis could be used to investigate changes to protein content in solution and could rule out whether impaired detection is attributed to loss of IgG-binding epitopes.

### 4.1. Specific Allergens Are Biomarkers for Contamination Assessments

The results of this study demonstrate that specific allergens make excellent biomarkers in allergen contamination assessments when paired with optimised extraction methods, providing reassurance that single, specific allergen analytical targets are sufficient for contamination detection. Several allergens were extracted in high abundance relative to total protein, as determined by Kjeldahl (up to 60%), and were readily detectable at 10 ppm in challenging food samples. Putting these results into context of VITAL 4.0 reference doses for precautionary allergen labelling, the ability to detect 10 ppm allergenic food is sufficient to cover VITAL 4.0 reference doses in 100 g portion size for all specified allergens [69,70]. Several allergens were quantified in the 10 ppm samples far higher than the analytical method limit of detection (see Supplementary Table S8). For example, Gal d 2 quantified > 4000-fold above the LOD in 10 ppm biscuit dough. This suggests that optimised extraction methods and analysis can detect total protein levels considerably lower than 10 ppm, making them suitable for identifying VITAL 4.0 action levels in larger portion sizes. Future studies should aim to investigate detection of lower levels of allergen, e.g., 1 ppm or lower.

It is noteworthy that shrimp tropomyosin, celery Api g 1, and soy Gly m 5 proved challenging to detect at 10–100 ppm doses in chocolate dessert and baked matrices. For these allergen sources, alternative allergen-specific analytical targets could be pursued. In the case of soy, 11 s globulin Gly m 6 could be a more suitable target for assay development due to greater heat resistance and abundance than Gly m 5 [71]. 11 s globulins Cor a 9 and Pru du 6 made for excellent markers of contamination in the current study due to their high abundance. Similarly, for celery detection, whilst Api g 4 is considered a minor allergen in terms of lower sensitisation prevalence compared to Api g 1 (75% vs. 42%) [72], this protein could make for a better analytical target in contamination assessments due to being more heat-stable [73]. As Api g 1 is a major allergen, immunoassay measurements with the methodology developed herein may be better suited to potency assessments of foods associated with the treatment and or prevention of allergy, rather than contamination assessments. Theoretically, shrimp tropomyosin should make for an excellent biomarker in crustacean contamination due to abundance and heat-stable properties. Whilst it is disappointing that in the current study, poor recoveries were observed at low doses, the methodology appears capable of detecting VITAL threshold reference dose of 200 mg shrimp protein in foods with a portion size of at least 500 g, even in the baked biscuit sample.

Whilst the methods for measuring Gly m 5, STM, and Api g 1 presented herein are not the most practical in identifying contamination, they still have potential use along with the other assays covered in potency and bioavailability assessments in foods associated with the diagnosis or treatment of allergy.

### 4.2. Specific Allergen Assays for Analysis and Standardisation of Foods Associated with the Diagnosis and Treatment of Allergies

This study outlines extraction and analytical methodology for measurement of clinically relevant allergens in foods, which could have major implications in improving the efficacy and safety of foods associated with the treatment and/or prevention of allergy. OFCs and OIT remain the current gold standard for food allergy diagnosis and treatment. In general, OFCs and OIT are conducted using commercially available foods that are not intended for medical purposes. Whilst the clinical approach is standardised, there is evidence to suggest that the foods used themselves could benefit from additional analysis and standardisation.

A recent study by Casale et al. found commercial foods used for peanut OFCs and OIT presented wide variations in specific allergen load between product types, but more concerningly, between batches. Batch–batch variation in specific allergen content in foods used for OFC may have implications akin to those recently observed in allergy skin prick tests in that, on occasion, false negatives may be observed depending on the dosing level used [74,75]. Another study investigated the allergen profile of milk muffins used for OFC and found that specific allergen levels varied considerably between recommended recipes, batches of preparation, and also within a single muffin itself (i.e., middle vs. top/bottom of baked muffin) [38]. The predominant finding from this study was that specific allergens presented varying risk due to differences in heat stability.

Work is being carried out to develop commercially available OFCs [76]. The chocolate dessert sample analysed herein is based on the Europrevall OFC matrix [44], and results demonstrate that optimised analytical methods for detecting clinically relevant allergens in an established OFC is feasible. The methodology developed herein shows scope not only in research studies but also has a place in standardising commercially available OFCs and OIT products with respect to specific allergen content.

### 4.3. Limitations and Future Work

Whilst the current study has analysed and optimised based on two matrices that pose challenges including presence of chocolate, high fat content (>20% for biscuit), and thermal processing from baking, a limitation of the current study is the number of matrices tested. Future studies could apply the optimised buffers to analyse a larger repertoire of matrices. Other frequently used matrices in food allergen detection method validations are ice creams, soups, sausages, and pasta, which may present additional challenges (e.g., impact of high salt content) [58,77]. Future studies may also wish to investigate the impacts of alternative food processing methods on allergen recovery, including but not limited to boiling, autoclaving to simulate canning/retorting (pressure and heating ~120 °C), frying, enzymatic treatment, and fermentation (e.g., cheeses, yoghurts, soy products), since these also have been demonstrated to impact allergenicity [68].

Results of this study demonstrate the challenges of identifying a single, optimal method of food allergen extraction spanning all allergenic proteins and matrix types. This is arguably a limitation of multiplex analytical methods. However, using appropriate buffer selection, all 14 major allergens may be analysed on a single multiplex run with enhanced efficiency compared to ELISA.

Two commercial chocolate-based food reference kits are available from LGC for food allergen analysis, together providing a reference sample for egg, milk, tree nuts, and peanut [78]. LGC kit analytical data presents poor recoveries (approximately 20% or less) for hazelnut and walnut from the incurred food matrix by a commercial ELISA kit. The chocolate dessert matrix developed in the current study is based on the same Europrevall matrix, yet matrix recoveries of 83–93% were observed in the present study for respective allergens from hazelnut and walnut sources, suggesting a strong extraction methodology has been developed. Future studies may wish to analyse this kit with the optimised extraction methods to corroborate the findings and may also provide supporting data for the reference kit preparation in terms of specific allergen content.

## 5. Conclusions

The present study underscores the significance of optimising allergen extraction protocols in complex food matrices. In addition, it provides enhanced understanding of the optimal extraction conditions for specific allergenic proteins from both source materials and complex food matrices. Such data will be imperative in developing robust contamination screening, characterisations of foods associated with the diagnosis, and treatment of allergies and as analytical reference materials.

## Figures and Tables

**Table 1:** Food extraction buffer formulations. PBS = phosphate-buffered saline. SDS = sodium dodecyl sulphate. NaCl = sodium chloride. PVP = polyvinylpyrrolidone-10. BSA = bovine serum albumin. NFDM = non-fat dry milk.

Buffer Identifier	Formulation
A	PBS, 2% Tween-20, 1 M NaCl, pH 7.4
B	PBS, 2% Tween-20, 1 M NaCl, 10% fish gelatine pH 7.4
C	0.1 M Tris, 1% SDS, 0.1 M sodium sulphite, pH 8.5
D	0.05 M sodium carbonate/sodium bicarbonate, 10% fish gelatine, pH 9.6
E	0.05 M Tris, 0.2 M NaCl, 10% fish gelatine, pH 8.3
F	0.1 M ammonium carbonate, pH 9.0
G	0.1 M ammonium carbonate, 10% fish gelatine pH 9.0
I	PBS, 2% Tween-20, 1 M NaCl, 0.25% BSA, 1% PVP, pH 7.4
J	PBS, 2% Tween-20, 1 M NaCl, 10% fish gelatine, 1% PVP pH 7.4
K	PBS, 2% Tween-20, 1 M NaCl, 2.5% NFDM, 1% PVP, pH 7.4

PBS: InBio, Cardiff, UK; Tween-20: Fisher Scientific, Leicester, UK; NaCl: VWR International, Leuven, Belgium; Fish Gelatine: Sigma-Aldrich, Gillingham, UK; Tris: Fisher Scientific, Leicester, UK; SDS: Thermo Scientific, Heysham, UK; Sodium sulphite: Thermo Scientific, Heysham, UK; Sodium carbonate: Fisher Scientific, Leicester, UK; Sodium bicarbonate: Fisher Scientific, Leicester, UK; Ammonium carbonate: Fisher Scientific, Leicester, UK; BSA: Sigma-Aldrich, Gillingham, UK; PVP: Sigma-Aldrich, Gillingham, UK; NFDM: Sigma-Aldrich, Gillingham, UK.

**Table 2:** Allergen source material extraction optimisation. Results reported as microgram of specific allergen per gram of source material spike mix (ug/g)^SMM^ ± coefficient of variation for triplicate extracts. Results converted to microgram of specific allergen per gram of total source material (ug/g)^T^ (see Supplementary Table S3). % Recovery^†^ ([observed/expected × 100]) and significance^∆^ (One-way Anova with Dunnett’s multiple comparison test) are relative to the extraction method with highest specific allergen yield for each allergen. Results in **bold** indicate buffer that yielded the highest amount of allergen. NS = Not significant. ND = Not determined. * *p* ≤ 0.05, ** *p* ≤ 0.01, *** *p* ≤ 0.001, **** *p* ≤0.0001. **^◊^** Sample extracted at 37 °C.

	Extraction Method									
	A	B	C	D	E	F	G	I	J	K
Pru du 6 (ug/g)^SMM^	**10,137 (±6%)**	9741 (±8%)	3355 (±13%)	8625 (±13%)	8613 (±6%)	7722 (±10%)	6574 (±7%)	8573 (±9%)	6875 (±4%)	8276 (±15%)
Pru du 6 (ug/g)^T^	**219,508**	210,946	72,653	186,765	186,525	167,230	142,369	185,653	148,888	179,207
% Recovery^†^	**100**	96	33	85	85	76	65	85	68	82
Significance^∆^	**-**	NS	****	NS	NS	**	****	NS	***	*
Jug r 1 (ug/g)^SMM^	**478 (±11%)**	416 (±11%)	2 (±23%)	258 (±6%)	307 (±3%)	244 (±4%)	252 (±14%)	422 (±18%)	370 (±4%)	411 (±14%)
Jug r 1 (ug/g)^T^	**8841**	7704	38	4776	5672	4514	4671	7800	6851	7603
% Recovery^†^	**100**	87	0.1	54	64	51	53	88	77	86
Significance^∆^	**-**	NS	****	****	***	****	****	NS	*	NS
Cor a 9 (ug/g)^SMM^	11,668 (±29%)	10,830 (±28%)	910 (±17%)	11,683 (±14%)	11,966 (±5%)	**12,883 (±6%)**	10,521 (±7%)	10,800 (±14%)	11,520 (±22%)	10,913 (±9%)
Cor a 9 (ug/g)^T^	78,813	73,149	6148	78,910	80,826	**87,019**	71,060	72,950	77,811	73,714
% Recovery^†^	91	84	7	91	93	**100**	82	84	89	85
Significance^∆^	NS	NS	****	NS	NS	**-**	NS	NS	NS	NS
Ana o 3 (ug/g)^SMM^	**2485 (±12%)**	2350 (±22%)	106 (±23%)	2320 (±29%)	1946 (±16%)	1905 (±6%)	1768 (±10%)	2155 (±23%)	1718 (±9%)	2430 (±31%)
Ana o 3 (ug/g)^T^	**21,564**	20,398	917	20,136	16,892	16,535	15,344	18,703	14,912	21,090
% Recovery^†^	**100**	95	4	93	78	77	71	87	69	98
Significance^∆^	**-**	NS	****	NS	NS	NS	NS	NS	NS	NS
Ses i 1 (ug/g)^SMM^	1124 (±9%)	1148 (±16%)	1 (±4%)	815 (±26%)	**1188 (±21%)**	451 (±3%)	536 (±9%)	977 (±15%)	651 (±3%)	980 (±14%)
Ses i 1 (ug/g)^T^	20,885	21,327	28	15,145	**22,081**	8389	9955	18,162	12,091	18,209
% Recovery^†^	95	97	0.1	69	**100**	38	45	82	55	82
Significance^∆^	NS	NS	****	*	**-**	****	****	NS	***	NS
Ara h 3 (ug/g)^SMM^	**772 (±7%)**	650 (±8%)	3 (±20%)	509 (±10%)	223 (±5%)	678 (±10%)	501 (±9%)	727 (±6%)	437 (±7%)	742 (±10%)
Ara h 3 (ug/g)^T^	**17,166**	14,454	68	11,320	4964	15,071	11,147	16,163	9718	16,483
% Recovery^†^	**100**	84	0.4	66	29	88	65	94	57	96
Significance^∆^	**-**	*	****	****	****	NS	****	ns	****	NS
Ara h 6 (ug/g)^SMM^	**213 (±6%)**	213(±10%)	1(±48%)	193 (±13%)	174 (±7%)	170 (±14%)	169 (±12%)	198 (±7%)	157 (±4%)	198 (±12%)
Ara h 6 (ug/g)^T^	**4745**	4728	33	4286	3874	3786	3747	4394	3483	4398
% Recovery^†^	**100**	100	0.7	90	82	80	79	93	73	93
Significance^∆^	**-**	NS	****	NS	NS	*	*	NS	**	NS
Gal d 1 (ug/g)^SMM^	1275 (±6%)	1305 (±7%)	114 (±10%)	**1383 (±3%)**	1177 (±19%)	1049 (±10%)	1055 (±8%)	1047 (±6%)	755 (±28%)	821 (±9%)
Gal d 1 (ug/g)^T^	41,816	42,784	3734	**45,359**	38,592	34,381	34,598	34,324	24,760	26,924
% Recovery^†^	92	94	8	**100**	85	76	76	76	55	59
Significance^∆^	NS	NS	****	**-**	NS	*	*	*	****	****
Gal d 2 (ug/g)^SMM^	**11,794 (±4%)**	11,346 (±15%)	12 (±10%)	10,879 (±20%)	10,268 (±11%)	7948 (±8%)	7370 (±15%)	8939 (±13%)	8023 (±3%)	8169 (±9%)
Gal d 2 (ug/g)^T^	**386,721**	372,022	392	356,726	336,676	260,619	241,651	293,104	263,077	267,855
% Recovery^†^	**100**	96	0.1	92	87	67	62	76	68	69
Significance^∆^	**-**	NS	****	NS	NS	**	***	*	**	**
Bos d 5 (ug/g)^SMM^	**701 (±20%)**	699 (±3%)	18 (±33%)	338 (±5%)	467 (±15%)	367 (±13%)	382 (±20%)	602 (±20%)	645 (±3%)	ND
Bos d 5 (ug/g)^T^	**9357**	9327	246	4517	6232	4892	5103	8035	8601	ND
% Recovery^†^	**100**	100	3	48	67	52	55	86	92	-
Significance^∆^	**-**	NS	****	****	**	***	***	NS	NS	
Bos d 11 (ug/g)^SMM^	2424 (±16%)	222 (±21%)	4827 (±7%)	**7504 (±16%)**	2699 (±8%)	819 (±7%)	921 (±16%)	ND	452 (±11%)	ND
Bos d 11 (ug/g)^T^	28,578	2957	64,410	**103,415**	36,019	10,934	12,288	ND	129	ND
% Recovery^†^	32	3	64	**100**	36	11	12	-	6	-
Significance^∆^	****	****	****	**-**	****	****	****		****	
STM (ug/g)^SMM^	29 (±11%)	28 (±3%)	45 (±17%)	42 (±11%)	**46 (±4%)**	42 (±6%)	33 (±8%)	18 (±28%)	19 (±6%)	19 (±16%)
STM (ug/g)^T^	776	749	1191	1117	**1212**	1127	870	480	498	494
% Recovery^†^	64	62	98	92	**100**	93	72	40	41	41
Significance^∆^	***	***	NS	NS		NS	**	****	****	****
Gly m 5 (ug/g)^SMM^	**2636 (±7%)**	2179 (±9%)	1825 (±7%)	1039 (±6%)	635 (±2%)	1665 (±25%)	1055 (±5%)	2311 (±2%)	1366 (±10%)	2279 (±10%)
Gly m 5 (ug/g)^T^	**55,675**	46,037	38,558	21,940	13,414	35,176	22,283	48,808	28,848	48,147
% Recovery^†^	**100**	83	69	39	24	63	40	88	52	86
Significance^∆^	**-**	*	***	****	****	****	****	NS	****	NS
Api g 1 (ug/g)^SMM^	20 (±37%)	20 (±18%)	31 (±31%)	8 (±12%)	46 (±28%)	19 (±9%)	9(±11%)	32 (±7%)	28 (±63%)	20 (±20%)
Api g 1 (ug/g)^T^	86	90	136	34	201	83	39	140	124	89
Api g 1 (ug/g)^SMM^ 37 °C^◊^	**75 (±8%)**	-	-	-	32 (±1%)	-	-	-	73 (±25%)	-
Api g 1 (ug/g)^T^ 37 °C^◊^	**330**				141				320	
% Recovery^†^	**100**				43				97	
Significance^∆^	**-**				*				NS	

**Table 3:** Optimised allergen detection from source materials. ^‡^ Total protein content of foods determined by Kjeldahl analysis using the standard conversion factor of ×6.25, unless otherwise stated. Specific Food and Agriculture Organisation of the United Nations conversion factors used for almond; 5.18 *^α^*, walnut/hazelnut/cashew/sesame; 5.3 *^β^*, peanut; 5.46 *^γ^,* cow’s milk; 6.38 *^δ^* and soy; 5.71 *^ε^* [57]. ^†^ Comparable results obtained by use of alternative extraction buffers (to within 10% highest value, no significant difference). * Api g 1 extracted at 37 °C.

Source Material	Total Protein (Kjeldahl, µg/g) ^‡^	Optimised Specific Allergen Content (µg/g)	Extraction Buffer	% Extracted Specific Allergen Relative to Total Protein
Almond Flour	438,435 *^α^*	Pru du 6	A (B ^†^)	50
		219,508		
Walnut Flour	383,296 *^β^*	Jug r 1	A	2
		8841		
Hazelnut Flour	139,920 *^β^*	Cor a 9	F (A/D/E ^†^)	62
		87,019		
Cashew Flour	179,776 *^β^*	Ana o 3	A (B/D ^†^)	12
		21,564		
Sesame Flour	384,992 * ^β^*	Ses i 1	E (A/B ^†^)	6
		22,081		
Light Roast Peanut Flour	474,364 *^γ^*	Ara h 3	A (I/K ^†^)	4
		17,166		
		Ara h 6	A (B/D/I/K ^†^)	1
		4745		
Egg Powder	801,000	Gal d 1	D (A/B ^†^)	6
		45,359		
		Gal d 2	A (/B/D/I/K ^†^)	48
		386,721		
Skim Milk Powder	332,780 *^δ^*	Bos d 5	A (B/J ^†^)	3
		9357		
		Bos d 11	D	30
		100,139		
Soybean Flour	471,417 *^ε^*	Gly m 5	A	12
		55,675		
Celeriac Powder	108,000	Api g 1	A * (J ^†^)	0.2
		330		
Shrimp Powder	648,000	Tropomyosin	E (C/D/F ^†^)	0.2
		1128		

**Table 4:** Incurred matrix recovery using optimised extraction buffers. <LOD = result below method limit of detection, no recovery value assigned. (+) denotes unexpected positive (0.012 µg/g/0.007 µg/g in biscuit dough/baked biscuit, respectively), no recovery value assigned, and value subtracted from incurred samples. Specific allergen measurements recorded in Supplementary Information Table S5.

Matrix		% Recovery from Incurred Matrix													
		Egg		Peanut		Cow’s Milk		Tree Nuts				Sesame	Shrimp	Celery	Soy
		Gal d 1	Gal d 2	Ara h 3	Ara h 6	Bos d 5	Bos d 11	Pru du 6	Ana o 3	Jug r 1	Cor a 9	Ses i 1	STM	Api g 1	Gly m 5
	Extraction Buffer:	J	J	D/J	D/J	J	D	J	J	J	J	J	D	J	J
Biscuit Dough	0 ppm	<LOD	<LOD	<LOD	<LOD	<LOD	<LOD	(+)	<LOD	<LOD	<LOD	<LOD	<LOD	<LOD	<LOD
	10 ppm	82	90	90/123	91/93	88	78	104	108	118	95	96	35	<LOD	69
	100 ppm	67	93	135/142	105/109	71	111	95	110	103	121	87	90	52	94
	1000 ppm	69	80	117/113	83/82	89	95	103	96	106	141	88	85	86	89
	Ave %	73	88	114/126	93	83	95	101	105	109	119	90	70	69	89
Baked Biscuit	0 ppm	<LOD	<LOD	<LOD	<LOD	<LOD	<LOD	(+)	<LOD	<LOD	<LOD	<LOD	<LOD	<LOD	<LOD
	10 ppm	<LOD	0	72/109	64/50	1	28	63	32	51	61	55	*<*LOD	<LOD	<LOD
	100 ppm	<LOD	0	51/77	48/45	1	24	48	28	64	87	44	5	<LOD	17
	1000 ppm	2	2	74/89	57/54	12	71	64	45	82	75	50	18	<1	30
	Ave %	2	1	66/92	56/50	5	41	58	35	66	74	50	11	<1	24
Chocolate Dessert	0 ppm	<LOD	<LOD	<LOD	<LOD	<LOD	<LOD	<LOD	<LOD	<LOD	<LOD	<LOD	<LOD	<LOD	<LOD
	10 ppm	52	74	63	47/68	52	49	71	93	101	73	88	16	<LOD	1
	100 ppm	59	84	53	64/52	44	34	51	64	83	80	80	66	56	14
	1000 ppm	60	66	70	58/49	55	74	65	70	96	97	65	91	79	39
	Ave %	60	75	62	56/57	50	52	63	76	93	83	78	58	67	22

## Data Availability

The original contributions presented in this study are included in the article/Supplementary Materials. Further inquiries can be directed to the corresponding author.

## Supplementary Materials

The following supporting information can be downloaded at: https://www.mdpi.com/article/10.3390/foods14203501/s1, Section S1: Review of Extraction buffer additives; Section S2: Preparation of allergen incurred foods; Table S1: Chocolate Dessert (CD) matrix ingredients; Table S2: Biscuit matrix ingredients; Table S3: Source material weights for incurring 1000 ppm matrices. Total protein (K) determined by nitrogen content, Kjeldahl analysis, 6.25× conversion factor; Section S3: Calculation of incurred matrix recovery example calculation; Section S4: Api g 1 extraction temperature assessment; Table S4: Extraction temperature assessment for Api g 1; Table S5: Specific allergen measurements using optimised extraction buffers; Table S6: 10ppm baked biscuit Bos d 11 quantification interference; Table S7: 10ppm baked biscuit dilution linearity extracted in buffer containing 2% Tween-20, example given Ara h 3; Table S8: Lowest level of detectable allergen relative to method limit of detection (LOD). References [79,80,81,82,83] are cited in the Supplementary Materials.

## Appendix A. Allergen Source Materials

Allergen source materials were identified from commercially available commodities with a view to obtain materials in a relevant, yet dispersible form, contain relevant allergenic protein with the allergens dispersed homogeneously throughout the material, and be free of unintended contamination. Source materials of commercial origin were selected based on the approach from Holcombe et al. [78]. They were identified on the following criteria: (i) free of ‘may contain’ unintended allergen contamination labelling, (ii) in a powdered/flour form, and (iii) in a relevant form, determined from use of a comparable material in previous reference materials, in clinical food challenges, and/or availability as a commercial food [1,78,84]. See Table A1 for identified materials.

**Table A1:** Allergen source materials. Processing notes obtained from product packaging or communication with supplier, unless otherwise stated.

Sample	Supplier	Item	Batch	Processing Notes
Egg Powder	Sigma-Aldrich (St. Louis, MO, USA)	Egg white powder EO500-1 kg	SLCG7215	57 °C drying
Milk Powder	Sigma-Aldrich (St. Louis, MO, USA)	Skimmed milk powder 70166-500 g	BCCH4990	72 °C pasteurisation and drying
Peanut Flour	Golden Peanut Company (Alpharetta, GA, USA)	Light roast peanut flour 12% Fat	119FA28318	Light dry roasted, defatted by pressing [85]. Light roasting = 121 °C for 11 min, then 157–166 °C for 14 min [86].
Soy Flour	Sigma-Aldrich (St. Louis, MO, USA)	Soybean flour Type 1S9633-500 g	SLCF4138	‘Not roasted, minimal heat treatment’.
Cashew Flour	Beyond the nut (Benin, West Africa)	Organic cashew flour	221118	Raw, ground
Walnut Flour	Hortus Verdi (Bihor, Romania)	Walnut protein flour	05/24_011	Raw, partially defatted by cold pressing, ground
Almond Flour	Sukrin (Lillestrøm, Norway)	Defatted almond flour	112390	Raw, defatted, ground
Hazelnut Flour	Bulgarian Nuts Premium (Mezhden, Bulgaria)	Hazelnut flour from ground blanched hazelnut	L20032023	Blanched, ground
Sesame Powder	Sukrin (Lillestrøm, Norway)	Sesame seed flour	111940	Dehusked, defatted, ground
Mustard Powder	Colmans (Norwich, UK)	Colman’s Mustard Powder, double superfine	L3012DM9715:27	Information not provided.
Celeriac (Celery root) Powder	Vehgro (Hengelo, Netherlands)	Celeriac ground organic powder	22144526	Dried < 90 °C, ground
Shrimp	Baracel (Market Drayton, UK)	Shrimp powder (cooked) 37411	100626.12	Steam cooked 100–140 °C 35 min, dried 110–120 °C 90 min
Salmon Powder	AABaits (Birkenhead, UK)	Salmon fishmeal	N/A	Information not provided.

## Appendix B. Extraction Temperature Verification; Pru du 6, Jug r 1, Ses i 1, and Api g 1

Filep and Chapman previously investigated extraction of specific allergens Ana o 3, Ara h 3, Ara h 6, Bos d 5, Bos d 11, Cor a 9, Gal d 1, Gal d 2, Gly m 5, and shrimp tropomyosin from source materials [8]. Extractions at 60 °C demonstrated a greater yield in detectable allergen, or at least no negative impact over extraction at RT. No data could be found in the literature on the impact of 60 °C extractions compared to RT for Pru du 6, Jug r 1, Ses i 1, and Api g 1, and so were assessed in a small-scale study, Table A1.

**Table A2:** Pru du 6, Jug r 1, Ses i 1, and Api g 1 extraction temperature assessment. Allergens were extracted from a source material mix at room temperature (RT), 37 °C, and 60 °C. Results expressed as microgram of specific allergen per gram of foodstuff (µg/g).

	Allergen Content (µg/g)			
Extraction Temperature	Almond, Pru du 6	Walnut, Jug r 1	Sesame, Ses i 1	Celery, Api g 1
RT	8602	378	900	72
37 °C	9343	382	1079	72
60 °C	10,326	501	1111	32
