# Supplementary material for: Optimising Extraction of Specific Food Allergens from Challenging Food Matrices for Immunoassay Quantification
Source: Foods. 2025 Oct 14;14(20):3501. doi: 10.3390/foods14203501 (PMC12563046; doi:10.3390/foods14203501)
Supplement: Supplementary file 1 [file foods-14-03501-s001.zip › foods-3855370-supplementary.pdf]

Optimising extraction of specific food allergens from challenging food matrices for immunoassay quantification – supplementary data

MD Bermingham[1,2]., RT Meredith[1]., H Mills[1]., S Maddocks[2]., MD Chapman[3]., JA Blaxland[2]., MA Oliver[1].

Affiliations:

[1]InBio, Cardiff, UK
[2]Cardiff Metropolitan University, Cardiff, UK.
[3]InBio, Charlottesville, VA. USA.
* Correspondence: mbermingham@inbio.com

S1. Review of Extraction buffer additives.

The addition of sodium chloride salt (NaCl) has been demonstrated to improve recovery of several allergens [1-3]. Whilst many allergens have been confirmed as readily water-soluble (e.g. 2S albumins), a significant number of allergen proteins require the presence of salts to maintain solubility [4,5]. The addition of salts increases the ionic strength of extraction buffers and can improve protein solubility [6].

Tween (Tween-20) is a non-ionic surfactant commonly used as an extraction buffer additive [2,7,8]. The non-ionic nature of Tween-20 does not impact native conformation like other anionic detergents, such as SDS, but has efficacy in breaking apart non-specific interactions [9]. Tween-20 has been reported to allow for the solubilisation of membrane proteins, and demonstrates capacity to breakdown lipid matrices [10]. Tween-20 has been implemented at 2% in the extraction of peanut allergens [11].

In samples containing polyphenolic compounds, such as chocolate, the addition of protein blocking agents has been demonstrated to improve the recovery of several allergens including bovine serum albumin (BSA), fish gelatine (FG) and non-fat dry milk (NFDM).

NFDM has demonstrated improved recoveries of peanut allergen, tree nut and soy allergens, used over a wide range of 0.25% up to 10% w/w [1,12-14]. The limitation of using NFDM is that the material will contain milk allergen proteins, and therefore not be suitable for the analysis of milk allergens in samples, which are the leading cause of food allergen recalls due to unintended allergen presence [15]. The use of refined bovine protein, such as BSA which is commonly used as a blocking agent in immunoassays presents as a more practical alternative.

In the late 1990's, the addition of 12.5% fish gelatine to a simple 0.1 M -Tris/0.4 M -NaCl buffer resulted in recovered peanut from chocolate increasing from 2-3% to ~60-90% [16]. Many following studies (and where published, commercial ELISA kit protocols) document improved recovery, or at least are reported suitable for peanut, hazelnut, almond, egg, sesame and milk (Bos d 5) at concentrations ranging 1-12.5% in various base buffer formulations including PBS, sodium carbonate and Tris/Tris-glycine [7,16-19]. Similarly to NFDM and milk analysis, the addition of fish gelatine could be problematic for the analysis of major fish allergens due to contamination. However, fish gelatine is obtained from fish skin, whereas the major fish allergens (e.g. parvalbumins) are present in fish muscle. Previous studies have found that processed commercial food-grade fish gelatine preparations contained undetectable (<0.02μg/g) levels of parvalbumin [20], thus meaning it may be feasible to

include fish gelatine in a universal extraction buffer, even when analysing for fish allergen, depending on the purity of fish gelatine used.

Non-protein blocking additives have also been used, including polyvinylpyrrolidone (PVP), which has been documented to improve the recovery of milk, soy and gluten allergen from polyphenol-containing matrices when used at 1-5% w/v [17,21,22].

The use of denaturing buffers has shown success in improving allergen recovery from processed samples. Theoretically, denaturing buffers work by breaking apart and or resolubilising insoluble allergen protein, whether that be due to denaturation, or formation of aggregates. Typically, these buffer systems make use of a chaotropic detergent (e.g. SDS) and a reducing agent (e.g. Beta-mercaptoethanol (BME)) in a relatively simple buffer base, such as tris. Due to toxicity, some labs prefer to not handle BME and may wish to use alternative reducing agents. A study by Ito et al investigated alternative denaturing buffers with reduced toxicological impacts and identified sodium sulfite to provide comparable recovery to BME [23]. It is important to note that such denaturing methods may not be feasible when immunoassay based methods target conformational epitopes, and likely will require generation of antibodies targeting denatured protein or linearised protein sequences [24,25].

Extraction buffer formulations and additives have been used in various combinations, which likely account for the variation in amount of each additive used between sources. In general, a consensus optimal extraction method spanning all allergen proteins from all sources remains elusive [26,27]."

S2. Preparation of allergen incurred foods

Ingredient screening

Ingredients used to prepare allergen-free matrices were chosen based on product labelling, e.g. no 'May Contain' labelling. Ingredients were confirmed to be allergen-free by immunoassay analysis. Materials were weighed out into new plastic-ware, and handled using disposable, single-use equipment. Mixing equipment was thoroughly cleaned with detergent prior to use.

Chocolate dessert

Chocolate dessert matrix was prepared according to Cochrane *et al* [28]. See Table S01 for ingredients. Dry ingredients were combined in a stainless-steel mixing bowl and mixed for 5 minutes. Mixing was performed using a Metcalfe SP-200 Heavy Duty Planetary Mixer set to speed 1. Tween-60 emulsifier was dissolved into maize oil at 37°C, mixing at 125 rpm for 10 minutes. The oil-emulsifier mix was subsequently added to dry ingredients and mixed for 5 minutes. Water was gradually added to the resulting paste, during continuous mixing for 5 minutes. Material from the sides and bottom of the mixing bowl was manually incorporated and mixed for 10 minutes. Material was transferred to a plastic container and frozen at -20°C prior to the incorporation of allergen.

Supplementary Table S1. Chocolate Dessert (CD) matrix ingredients.

CD ingredients	Supplier	Product details	Weight Used (g)
**Cold swelling starch**	Special Ingredients Ltd	ULTRATEX Instant thickener Batch K1135-0024	243
**Cocoa**	Dr. Oetker (UK) Ltd.	Fine Dark Cocoa Powder Batch L3179	413
**Caster sugar**	Silver Spoon, British Sugar plc	Caster Sugar Batch 3170FP	336
**Maize Oil**	Mazola (Edible Oils Ltd)	100% Pure corn oil Batch H3139	449
**Tween 60**	Merck Life Science UK Ltd	TWEEN 60 (Polysorbitan) #MKCP3449	11
**Rice flour**	Freee (Doves Farm Foods Ltd)	Freee Gluten Free Rice Flour #JC3201	45
**Water**	Welsh Water/Dwr Cymru Cardiff Supply	N/A	3500

Biscuit dough

Biscuit dough ingredients are outlined in Table S02. Initially, fats, sugars and flavourings were mixed for 10 minutes using a Metcalfe SP-200 Heavy Duty Planetary Mixer set to speed 1. Dry ingredients were subsequently added and mixed for an additional 10 minutes. Material from the sides and bottom of the mixing bowl was manually incorporated and mixed for 10 minutes. Material was transferred to a plastic container and frozen at -20°C prior to the incorporation of allergen.

Supplementary Table S2. Biscuit matrix ingredients.

Biscuit ingredients	Supplier	Product details	Weight Used (g)
**Gluten free rice flour**	Freee (Doves Farm Foods Ltd)	Gluten free rice flour 1KG Batch JC3201	1931
**Baking Powder**	Asda Stores Ltd	Baking Powder 170g Batch 3177	19
**Vegan butter**	Stork, Upfield Europe BV	Vegan alternative baking spread 500g Batch L33261076	1448
**Caster Sugar**	Silver Spoon, British Sugar plc	Caster Sugar Batch 3170FP	965
**Golden syrup**	Lyle's Golden Syrup, ASR Grp	Lyle Golden Syrup Pouring Batch P3156238	483
**Vanila extract**	Waitrose Ltd	Cooks' Ingredients Madagascan Vanilla Extract 38mL Batch L3242	8

Allergen source materials and spiking mix preparation

Source materials covering egg, cow's milk, peanut, soy, cashew, walnut, almond, hazelnut, sesame, mustard, celery, crustacea and fish can be identified in Appendix A of manuscript and Table S03.

Source materials were quantified for total protein content by Kjeldahl analysis Crude (soluble and insoluble) UKAS accredited laboratory. The methods indirectly determine protein content through measurement of nitrogen. Nitrogen content was extrapolated to total protein content by multiplication of a 6.25x conversion factor.

Allergen-free matrices were used as a base to prepare allergen-incurred samples. A 1000ppm high dose was incurred initially, whereby the required amount of each source material required to prepare 1kg of incurred matrix at 1000ppm was calculated (e.g. 3067mg skimmed milk powder with 32.6% protein content = 1000mg protein in 1kg matrix). See Table S03 for calculated spiking values.

Source materials were weighed to prepare a 'source material mix' (SMM).Weighing for the SMM preparation was a critical step in matrix preparation and required the highest level of precision. Materials were weighed using an analytical balance, calibrated with 1mg, 100mg, 1000mg and 200000mg E2 certified calibration weights on the day of weighing. Source materials were weighed to within ±1mg of target value (<0.1%). Source materials were weighed out onto Whatman low static weighing papers and combined in a polypropylene pot. The resulting source material mix was homogenised by vortex mixing at full speed for 2 minutes, placed on a roller mixer for 30 minutes at 60rpm and a final vortex for 2 minutes at full speed.

Supplementary Table S3. Source material weights for incurring 1000ppm matrices. Total protein (K) determined by nitrogen content, Kjeldahl analysis, 6.25x conversion factor.

Sample	Supplier	Item	µg/g Total Protein (K)	Target Concentration (µg/g)	Target mass food (g)	Amount of source material required (mg)	% Ingredient in spike mix
Egg Powder	Sigma	egg powder EO500-1kg	801,000	1000	1000	1248.4	3.0
Milk Powder	Sigma	skimmed milk powder 70166-500g	326,000	1000	1000	3067.5	7.5
Peanut Flour	Golden Peanut Company	Light roast peanut flour 12% Fat	543,000	1000	1000	1841.6	4.5
Soy Flour	Sigma	Soybean flour Type 1S9633-500g	516,000	1000	1000	1938.0	4.7
Cashew flour	Beyond the nut	Organic (raw) cashew flour	212,000	1000	1000	4717.0	11.5
Walnut flour	Hortus Verdi	(Raw) Walnut protein flour	452,000	1000	1000	2212.4	5.4
Almond flour	Sukrin	Defatted (Raw) Almond flour	529,000	1000	1000	1890.4	4.6
Hazelnut flour	Bulgarian Nuts Premium	Hazelnut flour	165,000	1000	1000	6060.6	14.8
Sesame powder	Sukrin	Dehusked and defatted (raw) sesame seed flour	454,000	1000	1000	2202.6	5.4
Mustard powder*	Colmans	Colmans Mustard Powder, double superfine	287,000	1000	1000	3484.3	8.5
Celeriac powder	Vehgro	Celeriac ground organic powder	108,000	1000	1000	9259.3	22.6
Shrimp Powder	Baracel	Shrimp powder 37411	648,000	1000	1000	1543.2	3.8
Salmon Powder*	AABaits	Salmon fishmeal	680,000	1000	1000	1470.6	3.6

***Note**: mustard and salmon source materials included in mix. Specific allergens from these were not analysed in the present study due to assay redevelopment.

1000ppm, 100ppm and 10ppm incurred matrix preparation

To form the 1000ppm matrices, approximately 500g of placebo matrix that had been defrosted and brought to room temperature was added to a stainless-steel mixing bowl, and allergen SMM carefully added on top. Additional placebo matrix was added to bring the total weight in the mixing bowl to 1000g. The matrix was homogenised using a Russell Hobbs GoCreate stand mixer on the lowest speed setting '1'. Matrix was mixed for 10 minutes, followed by manual incorporation of material from the side of the bowl and paddle mixer. This was repeated an additional 5 times to give a total of 1 hour mixing. 100ppm and 10ppm matrices were prepared by dilution of the 1000ppm or 100ppm 1-in-10 in placebo matrix (100g of 1000ppm to 900g placebo) and homogenised as previously described.

Baked biscuit preparation

Biscuit dough (placebo, 10, 100 and 1000ppm incurred) was chilled to 4°c, 40g portions were weighed out and rolled into spheres, stored at 4°C prior to baking. Biscuits were baked in a MONO DX DECK OVEN. Biscuits subjected to standard cooking were baked under recommendation by the Head of Baking at the ZERO2FIVE food industry centre, Cardiff, Wales, to model how a biscuit of this type would be cooked in a real-life scenario and therefore reflect how the item would be consumed. These conditions were 185°C for 15 minutes, where all biscuits were cooked on a single oven shelf.

Baked biscuits were homogenised by combining several biscuits within a single use Ziploc bag and crushing. The resulting crushed biscuits were ground using a Aigostar 300105KYI coffee grinder. Sample was ground on full speed for 30 seconds. Any material present on the sides or bound in large particles was distributed using a disposable spatula. This process was repeated a further 4 times for a total of 5 mixes.

S3. Calculation of incurred matrix recovery example calculation

Foods were incurred to 10ppm, 100ppm, and 1000ppm based on total protein determined by Kjedahl using the standard nitrogen conversion factor of 6.25.

1. Almond flour quantified by immunoassay to contain 219,508 microgram of Pru du 6 per gram of almond flour (µg/g, equivalent to parts per million [ppm]).
2. Almond flour was previously determined to contain 529,000 microgram of protein per gram of almond flour by Kjeldahl analysis.
3. *Calculate specific allergen:total protein ratio*
   a. *almond flour contains 219,508µg of Pru du 6 per 529,000µg of total protein.*
   b. 1µg almond protein contains 0.415µg Pru du 6
   c. Therefore, at defined levels of total protein*:*
      i. *1000ppm almond protein = 415*µg/g *Pru du 6.*
4. Incurred foods analysed by immunoassay to determine specific allergen content:
   1000ppm chocolate dessert contains 425µg/g Pru du 6
5. Calculate recovery (observed/expected)*100: ((425µg/g / 415µg/g)*100) = **103% Recovery**

S4. Api g 1 extraction temperature assessment.

Source material mix and 1000ppm incurred matrices were extracted in PBS, 2% Tween-20, 1M NaCl, pH 7.4 in a 1:10 sample ratio (e.g. 1g sample: 10mL extraction buffer), vortex mixed for 30 seconds to suspend material and incubated at either 37°C or 60°C for 15 minutes, shaking at 175rpm. Results are shown in Table S04.

Supplementary Table S4. Extraction temperature assessment for Api g 1. Results are average of n=2, or n=3◊ replicate extractions and reported as microgram of Api g 1 per gram of food (µg/g).

	Api g 1 (µg/g)		
	37°C	60°C	% Difference
**Source material spike mix**	72	32	-77
**1000ppm Biscuit Dough**	1.78	2.64◊	39
**1000ppm Baked Biscuit**	0.011	0.009◊	-20
**1000ppm Chocolate Dessert**	2.69	2.41◊	-11

Supplementary Table S5. Specific allergen measurements using optimised extraction buffers. Results reported as microgram per gram of food (µg/g). Spiking mix measured for µg/g specific allergen content†, multiplied up to expected specific allergen content in whole source material†† depending on % composition in spike mix (See table S03 for composition). Source material total protein content determined by Kjeldahl (µg/g)[nitrogen content x6.25]‡ used to calculate expected target value§ of specific allergen content at defined levels of total protein.

		**Specific allergen content (µg/g)**													
		**Egg**		**Peanut**		**Cow's Milk**		**Tree nuts**				**Sesame**	**Shrimp**	**Celery**	**Soy**
Matrix		**Gal d 1**	**Gal d 2**	**Ara h 3**	**Ara h 6**	**Bos d 5**	**Bos d 11**	**Pru du 6**	**Ana o 3**	**Jug r 1**	**Cor a 9**	**Ses i 1**	**TPM**	**Api g 1**	**Gly m 5**
	**Extraction buffer:**	**J**	**J**	**D/J***	**D/J***	**J**	**D**	**J**	**J**	**J**	**J**	**J**	**D**	**J**	**J**
Source Material	Spiking mix†	1383	11794	772	213	701	7504	10137	2485	478	12883	1188	46	75	2636
	Source material††	45359	386721	17166	4745	9357	100139	219508	21564	8841	87019	22081	1212	330	55675
	Source total protein‡	801000‡		543000‡		326000‡		529000‡	212000‡	452000‡	165000‡	454000‡	648000‡	108000‡	516000‡
Expected Target Value§	**10ppm**	0.57	4.8	0.32	0.087	0.29	3.1	4.1	1.0	0.20	5.3	0.49	0.019	0.031	1.1
	**100ppm**	5.7	48	3.2	0.87	2.9	31	41	10	2.0	53	4.9	0.19	0.31	11
	**1000ppm**	57	483	32	8.7	29	307	415	102	20	527	49	1.9	3.1	108
Biscuit Dough	0ppm	*<0.3125*	*<0.002*	*<0.0048*	*<0.0004*	*<0.002*	*<0.002*	*0.012*	*<0.0008*	*<0.0008*	*<0.020*	*<0.001*	*<0.002*	*<0.0312*	*<0.078*
	10ppm	0.46	4.4	0.28/0.39	0.080/0.081	0.25	2.4	4.3	1.1	0.23	5.0	0.47	0.006	*<0.0312*	0.74
	100ppm	3.8	45	4.3/4.5	0.92/0.95	2.0	34	39	11	2.0	64	4.2	0.17	0.16	10
	1000ppm	39	385	37/36	7.2/7.2	26	292	426	98	21	741	43	1.6	2.6	113
Baked Biscuit	0ppm	*<0.3125*	*<0.002*	*<0.0048*	*<0.0004*	*<0.002*	*<0.002*	*0.007*	*<0.0008*	*<0.0008*	*<0.020*	*<0.001*	*<0.002*	*<0.0312*	*<0.078*
	10ppm	*<0.3125*	0.003	0.23/0.35	0.056/0.043	0.003	0.86	2.6	0.33	0.10	3.2	0.27	*<0.002*	*<0.0312*	*<0.078*
	100ppm	*<0.3125*	0.074	1.6/2.4	0.42/0.39	0.030	7.4	20	2.9	1.3	46	2.2	0.010	<0.0078	1.9
	1000ppm	0.97	11	23/28	5.0/4.8	3.5	218	264	46	16	396	24	0.33	0.009	32
Chocolate Dessert	0ppm	*<0.3125*	*<0.002*	*<0.0048*	*<0.0004*	*<0.002*	*<0.002*	*<0.0048*	*<0.0008*	*<0.0008*	*<0.020*	*<0.001*	*<0.002*	*<0.0312*	*<0.078*
	10ppm	0.29	3.6	0.20	0.041/0.060	0.15	1.5	3.0	0.95	0.20	3.8	0.43	0.003	*<0.0312*	0.13
	100ppm	3.3	40	1.7	0.56/0.46	1.3	11	21	6.5	1.6	42	3.9	0.12	0.17	1.5
	1000ppm	34	321	22	5.1/4.3	16	226	270	72	19	511	31	1.7	2.4	42

Supplementary Table S6. 10ppm baked biscuit Bos d 11 quantification interference. Results are expressed as concentration in well (conc) × sample dilution (dil), reported as nanograms of Bos d 11 per millilitre (ng/ml). Inconsistent agreement across sample dilutions was observed (coefficient of variation, CV%, 52–110%), preventing reliable quantification. These findings indicate assay interference likely attributable to the extraction buffer.

	**Extraction Method**	**Extraction replicate**	**Sample dil, conc x dil (ng/ml)**				**Avg conc x dil (ng/ml)**	**CV%**
			**1 / 2**	**1 / 8**	**1 / 32**	**1 / 128**		
**Plate 1**	**C**	**1**	6	17	117	160	75	**101**
	**C**	**2**	6	17	105	161	72	**102**
	**C**	**3**	6	17	133	142	75	**98**
			**1 / 4**	**1 / 16**	**1 / 64**	**1 / 256**		
**Plate 2**	**C**	**1**	16	52	102	196	91	**86**
	**C**	**2**	15	54	101	213	95	**90**
	**C**	**3**	15	44	107	297	116	**110**
			**1 / 10**	**1 / 40**	**1 / 160**	**1 / 640**		
**Plate 3**	**C**	**4**	29	87	181	308	151	**81**
	**C**	**5**	18	118	144	251	133	**72**
	**C**	**6**	***	106	153	288	182	**52**

******software could not calculate concentration due to insufficient bead count in well***

Supplementary Table S7. 10ppm baked biscuit dilution linearity extracted in buffer containing 2% Tween-20, example given Ara h 3*. Results expressed as concentration in well (conc) x sample dilution (dil), nanogram allergen per millilitre (ng/ml). Strong agreement between sample dilutions were observed (coefficient of variation (CV%) ranged 1-16%), meaning sample could be reliably quantified. These data suggest no interference from buffers containing 2% Tween-20 detergent when samples analysed as low as 1:2.

	**Sample dil, conc x dil (ng/ml*)**				
**Sample**	**1 / 2**	**1 / 8**	**1 / 32**	**Avg conc x dil (ng/ml)**	**%CV**
Baked biscuit, 10ppm rep 1	24	30	33	29	16
Baked biscuit, 10ppm rep 2	24	30	29	28	11
Baked biscuit, 10ppm rep 3	23	24	23	23	1

Supplementary Table S8. Lowest level of detectable allergen relative to method limit of detection (LOD). Unless otherwise specified results are from 10ppm incurred samples. *Denotes 100ppm samples, **denotes 1000ppm sample.

			Biscuit Dough		Chocolate Dessert		Baked Biscuit	
**Allergen**	**Specific Allergen**	**LOD (µg/g)**	**Allergen Content (µg/g)**	Fold higher	**Allergen Content (µg/g)**	Fold higher	**Allergen Content (µg/g)**	Fold higher
**Almond**	**Pru du 6**	0.0024	4.30	1792	2.96	1233	2.62	1090
**Walnut**	**Jug r 1**	0.0004	0.23	577	0.20	492	0.10	251
**Hazelnut**	**Cor a 9**	0.0078	5.02	644	3.84	492	3.20	411
**Cashew**	**Ana o 3**	0.0004	1.09	2736	0.95	2365	0.33	819
**Sesame**	**Ses i 1**	0.0010	0.47	469	0.43	430	0.27	268
**Peanut**	**Ara h 3**	0.0024	0.39	163	0.13	54	0.35	144
	**Ara h 6**	0.0002	0.08	406	0.06	299	0.04	217
**Egg**	**Gal d 1**	0.1563	0.46	3	0.29	2	0.97**	6
	**Gal d 2**	0.0010	4.36	4363	3.59	3588	0.00	3
**Cow's milk**	**Bos d 5**	0.0010	0.25	252	0.15	149	0.00	3
	**Bos d 11**	0.0010	2.39	2390	1.52	1516	0.86	856
**Soy**	**Gly m 5**	0.0781	0.08	10	0.02	2	0.12*	15
**Celery**	**Api g 1**	0.0039	0.16*	2	0.17**	2	0.01**	426
**Shrimp**	**STM**	0.0010	0.01	6	0.01	3	0.003*	10

References:

1. Vinton, R.; Chapman, M.D.; Pomes, A. Peanut allergen (ara h 1) detection in foods containing chocolate. *Journal of Allergy and Clinical Immunology* **2003**, *111*, S194, doi:http://dx.doi.org/10.1016/S0091-6749(03)80664-4.
2. Filep, S.; Chapman, M.D. Doses of Specific Allergens in Early Introduction Foods for Prevention of Food Allergy. *The Journal of Allergy and Clinical Immunology: In Practice* **2022**, *10*, 150-158.e153, doi:https://doi.org/10.1016/j.jaip.2021.02.051.
3. Alves, R.C.; Pimentel, F.B.; Nouws, H.P.A.; Silva, T.H.B.; Oliveira, M.B.P.P.; Delerue-Matos, C. Improving the extraction of Ara h 6 (a peanut allergen) from a chocolate-based matrix for immunosensing detection: Influence of time, temperature and additives. *Food Chemistry* **2017**, *218*, 242-248, doi:http://dx.doi.org/10.1016/j.foodchem.2016.09.085.
4. Guo, F.; Kothary, M.H.; Wang, Y.; Yu, X.; Howard, A.J.; Fu, T.J.; Zhang, Y.Z. Purification and crystallization of Cor a 9, a major hazelnut allergen. *Acta Crystallogr Sect F Struct Biol Cryst Commun* **2009**, *65*, 42-46, doi:10.1107/s1744309108039894.
5. Dramburg, S.; Hilger, C.; Santos, A.F.; de Las Vecillas, L.; Aalberse, R.C.; Acevedo, N.; Aglas, L.; Altmann, F.; Arruda, K.L.; Asero, R.; et al. EAACI Molecular Allergology User's Guide 2.0. *Pediatr Allergy Immunol* **2023**, *34 Suppl 28*, e13854, doi:10.1111/pai.13854.
6. Nugraha, R.; Ruethers, T.; Johnston, E.B.; Rolland, J.M.; O'Hehir, R.E.; Kamath, S.D.; Lopata, A.L. Effects of Extraction Buffer on the Solubility and Immunoreactivity of the Pacific Oyster Allergens. *Foods* **2021**, *10*, doi:10.3390/foods10020409.
7. Neogen. BioKits Egg Assay Kit - product insert. Edition 902072T. 2010. Accessed on: 10 July 2025. Available at: www.neogen.com/49b7fe/globalassets/pim/assets/original/10017/t201391-biokits-egg-assay_902072t_kitinsert.pdf.

8. Pomés, A.; Vinton, R.; Chapman, M.D. Peanut allergen (Ara h 1) detection in foods containing chocolate. *J Food Prot* **2004**, *67*, 793-798, doi:10.4315/0362-028x-67.4.793.
9. Chen, J.; Feng, Y.; Kong, B.; Xia, X.; Liu, Q. An eco-friendly extraction method for adsorbed proteins from emulsions stabilized by whey protein isolate by using Tween 20. *Colloids and Surfaces A: Physicochemical and Engineering Aspects* **2020**, *604*, 125332, doi:https://doi.org/10.1016/j.colsurfa.2020.125332.
10. Hjertén, S.; Johansson, K.-E. Selective solubilization with tween 20 of membrane proteins from Acholeplasma laidlawii. *Biochimica et Biophysica Acta (BBA) - Biomembranes* **1972**, *288*, 312-325, doi:https://doi.org/10.1016/0005-2736(72)90252-0.
11. Pomés, A.; Helm, R.M.; Bannon, G.A.; Burks, A.W.; Tsay, A.; Chapman, M.D. Monitoring peanut allergen in food products by measuring Ara h 1. *Journal of Allergy and Clinical Immunology* **2004**, *111*, 640-645, doi:10.1067/mai.2003.118.
12. Pei Wen Lim. Development Of An Enzyme-Linked Immunosorbent Assay (ELISA) For The Detection Of Pistachio Residues In Processed Foods. University of Nebraska - Lincoln, 2010.
13. Huet, A.C.; Paulus, M.; Henrottin, J.; Brossard, C.; Tranquet, O.; Bernard, H.; Pilolli, R.; Nitride, C.; Larré, C.; Adel-Patient, K.; et al. Development of incurred chocolate bars and broth powder with six fully characterised food allergens as test materials for food allergen analysis. *Anal Bioanal Chem* **2022**, *414*, 2553-2570, doi:10.1007/s00216-022-03912-z.
14. Alves, R.C.; Pimentel, F.B.; Nouws, H.P.A.; Silva, T.H.B.; Oliveira, M.; Delerue-Matos, C. Improving the extraction of Ara h 6 (a peanut allergen) from a chocolate-based matrix for immunosensing detection: Influence of time, temperature and additives. *Food Chem* **2017**, *218*, 242-248, doi:10.1016/j.foodchem.2016.09.085.
15. Yue, S.R.; Shrivastava, R.; Campbell, K.; Walker, M.J. Food allergen recalls in the United Kingdom: A critical analysis of reported recalls from 2016 to 2021. *Food Control* **2023**, *144*, 109375, doi:https://doi.org/10.1016/j.foodcont.2022.109375.
16. Keck-Gassenmeier, B.; Benet, S.; Rosa, C.; Hischenhuber, C. Determination of Peanut Traces in Food by a Commercially-available ELISA Test. *Food and Agricultural Immunology* **1999**, *11*, 243-250, doi:10.1080/09540109999762.
17. Nguyen, A.V.; Lee, D.; Williams, K.M.; Jackson, L.S.; Bedford, B.; Kwon, J.; Scholl, P.F.; Khuda, S.E. Effectiveness of antibody specific for heat-processed milk proteins and incurred calibrants for ELISA-based quantification of milk in dark chocolate matrices. *Food Control* **2021**, *123*, 107760, doi:https://doi.org/10.1016/j.foodcont.2020.107760.
18. Stephan, O.; Möller, N.; Lehmann, S.; Holzhauser, T.; Vieths, S. Development and validation of two dipstick type immunoassays for determination of trace amounts of peanut and hazelnut in processed foods. *European Food Research and Technology* **2002**, *215*, 431-436, doi:10.1007/s00217-002-0562-6.
19. Neogen. BioKits BLG Assay Kit - Kit Insert. Edition 902061Y. 2010. Accessed on: 12 July 2025. Available at: www.neogen.com/49e8dd/globalassets/pim/assets/original/10017/t201185-biokits-assay-kit-biokits-blg-assay_902061y_kitinsert.pdf.
20. Koppelman, S.J.; Nordlee, J.A.; Lee, P.W.; Happe, R.P.; Hessing, M.; Norland, R.; Manning, T.; Deschene, R.; De Jong, G.A.H.; Taylor, S.L. Parvalbumin in fish skin-derived gelatin: is there a risk for fish allergic consumers? *Food Additives & Contaminants: Part A* **2012**, *29*, 1347-1355, doi:10.1080/19440049.2012.698399.
21. Satsuki-Murakami, T.; Kudo, A.; Masayama, A.; Ki, M.; Yamano, T. An optimized extraction method for gluten analysis in cacao-containing products using an extraction buffer with polyvinylpyrrolidone. *Food Control* **2018**, *84*, 70-74, doi:https://doi.org/10.1016/j.foodcont.2017.07.025.
22. Lacorn, M.; Dubois, T.; Siebeneicher, S.; Weiss, T. Accurate and Sensitive Quantification of Soy Proteins in Raw and Processed Food by Sandwich ELISA. *Food Science and Technology* **2016**, *4*, 69-77, doi:10.13189/fst.2016.040404.
23. Ito, K.; Yamamoto, T.; Oyama, Y.; Tsuruma, R.; Saito, E.; Saito, Y.; Ozu, T.; Honjoh, T.; Adachi, R.; Sakai, S.; et al. Food allergen analysis for processed food using a novel extraction method to

eliminate harmful reagents for both ELISA and lateral-flow tests. *Anal Bioanal Chem* **2016**, *408*, 5973-5984, doi:10.1007/s00216-016-9438-7.
24. Jayasena, S.; Wijeratne, S.S.K.; Taylor, S.L.; Baumert, J.L. Improved extraction of peanut residues from a wheat flour matrix for immunochemical detection. *Food Chemistry* **2019**, *278*, 832-840, doi:https://doi.org/10.1016/j.foodchem.2018.11.123.
25. Watanabe, Y.; Aburatani, K.; Mizumura, T.; Sakai, M.; Muraoka, S.; Mamegosi, S.; Honjoh, T. Novel ELISA for the detection of raw and processed egg using extraction buffer containing a surfactant and a reducing agent. *Journal of Immunological Methods* **2005**, *300*, 115-123, doi:https://doi.org/10.1016/j.jim.2005.02.014.
26. Westphal, C.D.; Pereira, M.R.; Raybourne, R.B.; Williams, K.M. Evaluation of extraction buffers using the current approach of detecting multiple allergenic and nonallergenic proteins in food. *J AOAC Int* **2004**, *87*, 1458-1465.
27. Lacorn, M.; Immer, U. Chapter 9 - Enzyme-linked immunosorbent assays (ELISAs) for detecting allergens in food∗. In *Handbook of Food Allergen Detection and Control (Second Edition)*, Flanagan, S., Ed.; Woodhead Publishing: 2025; pp. 173-189.
28. Cochrane, S.A.; Salt Lj Fau - Wantling, E.; Wantling E Fau - Rogers, A.; Rogers A Fau - Coutts, J.; Coutts J Fau - Ballmer-Weber, B.K.; Ballmer-Weber Bk Fau - Fritsche, P.; Fritsche P Fau - Fernández-Rivas, M.; Fernández-Rivas M Fau - Reig, I.; Reig I Fau - Knulst, A.; Knulst A Fau - Le, T.M.; et al. Development of a standardized low-dose double-blind placebo-controlled challenge vehicle for the EuroPrevall project. *Allergy* **2012**, *67(1):107-13.*, doi:10.1111/j.1398-9995.2011.02715.x.
